# Malaria in rural Mozambique. Part I: Children attending the outpatient clinic

**DOI:** 10.1186/1475-2875-7-36

**Published:** 2008-02-26

**Authors:** Caterina Guinovart, Quique Bassat, Betuel Sigaúque, Pedro Aide, Jahit Sacarlal, Tacilta Nhampossa, Azucena Bardají, Ariel Nhacolo, Eusébio Macete, Inácio Mandomando, John J Aponte, Clara Menéndez, Pedro L Alonso

**Affiliations:** 1 Barcelona Center for International Health Research (CRESIB), Hospital Clínic/Institut d'Investigacions Biomèdiques August Pi i Sunyer, Universitat de Barcelona, Rosselló 132, E-08036 Barcelona, Spain; 2 Centro de Investigação em Saúde de Manhiça (CISM), Manhiça, CP 1929, Maputo, Mozambique; 3 Instituto Nacional de Saúde, Ministério de Saúde, Maputo, Mozambique; 4 Faculdade de Medicina, Universidade Eduardo Mondlane, Maputo, Mozambique; 5 Direcção Nacional de Saúde, Ministério de Saúde, Maputo, Mozambique

## Abstract

**Background:**

Malaria represents a huge burden for the health care services across Africa. Describing malaria attending health services contributes to quantify the burden and describe the epidemiology and clinical presentation.

**Methods:**

Retrospective analysis of data collected through the Manhiça morbidity surveillance system (Mozambique) on all paediatric visits (<15 years) to the outpatient clinic from June 2003 to May 2005. Age-specific minimum community-based incidence rates (MCBIRs) of malaria were calculated using demographic surveillance system data. Malaria was defined as fever or history of fever in the preceding 24 hours with asexual *Plasmodium falciparum *parasitaemia of any density in the blood smear.

**Results:**

A total of 94,941 outpatient visits were seen during the study period, of which 30.5% had malaria. Children younger than three years accounted for almost half of the total malaria cases and children aged ≥ 5 years represented 36.4% of the cases. Among children who presented with malaria, 56.7% had fever and among children who presented with fever or a history of fever only 37.2% had malaria. The geometric mean parasitaemia in malaria cases was 8582.2 parasites/μL, peaking in children aged two to three years. 13% of malaria cases had a PCV<25% and the mean PCV in malaria cases increased gradually with age, ranging from 27.8% in children aged 2–12 months to 34.4% in ≥ 5 years. The percentage of cases admitted or transferred showed a clear decreasing trend with age. MCBIRs of outpatient malaria per 1,000 child years at risk for the whole study period were of 394 in infants, 630 in children aged 1 to <5 years and 237 in children aged five years or more. A clustering of the cases was observed, whereby most children never had malaria, some had a few episodes and very few had many episodes.

**Conclusion:**

Preventive measures should be targeted at children younger than three years, as they carry the highest burden of malaria. Children aged 5–15 years represent around a third of the malaria cases and should also be included in control programmes. Concern should be raised about presumptive treatment of fever cases with artemisinin-combination therapies, as many children will, according to IMCI guidelines, receive treatment unnecessarily.

## Background

Malaria continues to be the first single cause of mortality in children in sub-Saharan Africa [1] and a major obstacle for the economic development in many countries [2]. Moreover, malaria represents between 25 and 40% of all outpatient visits to health centres in African malaria-endemic countries [1]. It is critical that control programmes implement available cost-effective control tools to decrease the burden of disease, especially among young children and pregnant women.

In Mozambique, as in most sub-Saharan African countries, the health management information system is weak [3]. Therefore, the Ministry of Health has incomplete and inaccurate data on the burden of disease of a given pathology, making it difficult for managers to develop evidence-based health policies. Also, few studies have been published on the malaria epidemiology in the country [4-7]. It is crucial that data on the incidence and age distribution of malaria is made available and is used to define and assess control programmes. Hospital surveillance data, despite being influenced by health-seeking behaviour and accessibility, are a valuable and often the only source of information on the most prevalent health problems of a community and their epidemiology.

Data are presented on the characteristics of malaria in children who attend the outpatient clinic of a rural hospital and minimum community-based incidence rates in a rural area of southern Mozambique. Data on children with malaria admitted to the same hospital are presented in a companion article [8].

## Methods

### Study site and population

The study area is located in Manhiça, Maputo Province, southern Mozambique. The area has been described in detail elsewhere [9]. There are two different seasons: a warm and rainy season and a cool dry season. The mean annual temperature is 22.5°C, ranging from a mean temperature of 25.5°C in January to 18.3°C in July. The annual rainfall was of 1286 mm in the year 2003, of 1165 mm in 2004 and of 687 in 2005.

Malaria transmission in the area, mainly caused by *Plasmodium falciparum*, is perennial with substantial seasonality and of moderate intensity. The average entomological inoculation rate (EIR) was of 38 infective bites per person per year in 2002, being *Anopheles funestus *the main vector [10,11].

Malaria control in the area is based on prompt treatment of cases, the coverage of insecticide-treated nets being <10% throughout the study period. The Manhiça District Hospital is the main health facility in the area, used for primary health care by the nearby population, and one of the two referral health centres for Manhiça District. It has a 110-bed inpatient ward, an outpatient clinic, a maternal and child health clinic with a small surgical room, and an emergency room [12]. There are two other peripheral health posts in the area, used only for primary health care. Both the hospital and the health posts are easily accessible and all outpatient consultations are free, except for a standard subsidized fee for the outpatient medication to be taken home.

The Manhiça Health Research Center (Centro de Investigação em Saúde de Manhiça, CISM) runs since 1996 a Demographic Surveillance System in the area [9] and a morbidity surveillance system at Manhiça District Hospital and other peripheral health posts in the area, including the Maragra Health Post, 20 km south of Manhiça District Hospital. The DSS covers Manhiça town and the surrounding villages, the Manhiça study area, with a total population under surveillance of around 80,000 inhabitants, 44% of which are under 15 years of age. The infant-mortality rate in the study area in 2005 was 77.5 per 1,000 live births, and the under-five mortality rate was 138.6 per 1,000 [13]. All children resident in the study area have a card with a permanent identification number issued by the DSS.

### Study design

Retrospective analysis of data collected through the Manhiça morbidity surveillance system. All paediatric visits (<15 years) to the outpatient clinic from 1^st ^of June 2003 to 31^st ^of May 2005 were analysed.

### Hospital surveillance system

A passive case detection system was established in 1996 to cover all paediatric (children aged 0–<15 years) outpatient and inpatient visits to Manhiça District Hospital and all outpatient visits to the Maragra Health Post. A standardized questionnaire, which includes personal and demographic data (including the permanent identification number) and clinical signs and symptoms, is completed for each child seen at the outpatient clinic. The clinician on duty, usually a medical agent (medical agents receive a clinical training of at least two years after secondary school), records the physical signs found on examination and the symptoms and duration of those as referred by the child's guardian. The axillary temperature is taken with an electronic thermometer and a finger-prick blood sample is collected from children who present fever (axillary temperature ≥ 37.5°C) or report a history of fever in the preceding 24 hours. Blood is collected into heparinized capillaries to measure the packed cell volume (PCV) and thin and thick blood smears are prepared to determine parasitaemia. Final diagnosis/es are recorded on the questionnaire by the clinician upon discharge or admission to the ward, after review of all signs, symptoms and laboratory results.

### Case management

Children seen at the outpatient clinic of Manhiça District Hospital are either sent home after diagnosis and treatment, or, if they fulfill severity criteria, are referred to the day-care unit of the hospital. The day-care unit was created by CISM to operate as an intermediate step between the outpatient clinic and the paediatric ward. Children seen at the outpatient clinic who fulfill severity criteria or need close monitoring are referred to the day-care unit, where they are reassessed by a more senior clinician, stabilized, and they receive initial treatment. This unit only works during the day and before it closes all children are either discharged, after a final diagnosis and treatment, or admitted to the wards. The referral hospital for Manhiça is the Maputo Central Hospital, where some of the children are transferred, being the unavailability of blood for transfusion the main reason.

Children seen at the Maragra Health Post fulfilling severity criteria are transferred to the Manhiça District Hospital, where they follow the standard circuit. Malaria cases were treated according to the Mozambican national guidelines. The first line treatment for ambulatory patients was chloroquine until it was changed in 2003 to sulphadoxine-pyrimethamine (SP) plus amodiaquine (AQ). In 2006 it was changed again to SP plus artesunate. During the study period the first line treatment was SP+AQ (parasitological efficacy was 92% in 2001–02 in Manhiça [14]) and Co-Artem^® ^was the second line treatment. Patients who were referred to the day-care unit and later to the wards received parenteral quinine for a minimum of six doses, followed by SP, or continued up to 21 doses if given as monotherapy.

### Laboratory methods

The PCV was measured using a microhaematocrit centrifuge and a Hawksley reader (Hawksley & Sons Ltd, Lancing, UK). Thin and thick blood films were read to quantify parasitaemia in the CISM laboratory according to standard procedures. Blood films were air-dried, Giemsa-stained, and examined using a light microscope fitted with a 10× oil immersion lens. Slides were declared negative only after 2000 leukocytes have been counted. Parasite numbers were converted to a count/μL by assuming a standard leukocyte count of 8000/μL.

### Definitions

Malaria was defined as fever (axillary temperature of ≥ 37.5°C) or a history of fever in the preceding 24 hours plus a *P. falciparum *asexual parasitaemia of any density. No age cut-off for the level of parasitaemia was used to increase the specificity of the definition, as this is the definition used at the outpatient clinic for all ages to diagnose malaria and administer antimalarials.

Mild anaemia was defined as a PCV between 25% and <33%, moderate anaemia as a PCV 15% to <25% and severe anaemia as a PCV<15%. Hyperpyrexia was defined as a measured axillary temperature of ≥ 39°C. The rainy season was defined as November to April and the dry season as May to October.

### Data management and statistical methods

Questionnaires were double entered into databases using a programme written in Fox Pro (Microsoft Corp., Seattle, WA, USA) at CISM. Statistical analyses were performed using Stata 9 (Stata Corp., College Station, TX, USA). The DSS permanent identification number, which is recorded on the morbidity questionnaires when children attend the hospital, was used to classify children as residents/non-residents in the study area. Occasionally, the mother/accompanying person fails to show the card when attending the hospital and the number is not recorded, thus the number of outpatient visits of children from the study area are underestimates.

Minimum community-based incidence rates (MCBIRs) were calculated as the age-specific yearly number of malaria cases visited at the outpatient clinic in children resident in the study area divided by the total child years at risk (CYAR) for that age group and year. CYAR were estimated from the DSS databases and children did not contribute to the numerator or denominator for a period of 28 days after each episode of malaria, when they were outside the study area or after death. Rate ratios and their p-values were estimated using the Mantel-Haenzel method to compare age-specific and year-specific rates.

Qualitative variables were compared using a χ^2 ^test or Fisher's exact test. Means of normally distributed variables were compared using the Student's t-test or ANOVA.

Geometric means of parasitaemia were compared using the Wilcoxon Rank sum test.

Parasitaemia results were missing for 1.8% and PCV results for 1.5% of the outpatients who had fever or a history of fever. Age was missing in 0.2% of the outpatients.

## Results

A total of 94,940 visits were seen at the outpatient clinic of Manhiça District Hospital and Maragra Health Post during the two year study period. Of these, 30.5% (28,963) had malaria, ranging from 34.1% in the first year of surveillance to 26.5% in the second year. Males accounted for 50.5% of the malaria cases.

**Figure 1:**
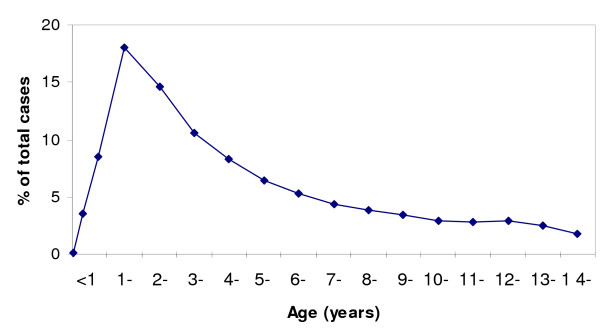
Age distribution of outpatient malaria cases.

**Figure 2:**
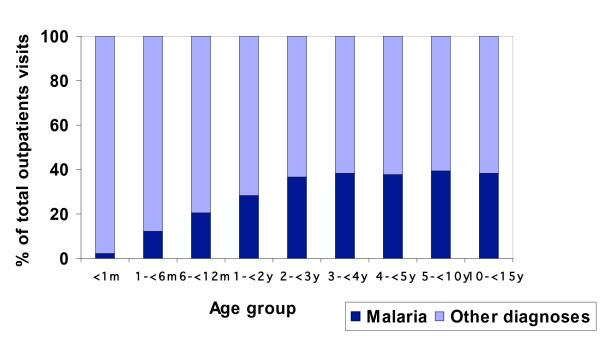
Relative contribution of malaria to the outpatient visits in children less than 15 years of age.

The age distribution of malaria cases is shown in Figure 1. Children younger than three years accounted for almost half of the total malaria cases, the highest number of cases being in children in their second year of life. Children aged five years or older represented 36.4% of the total malaria cases. Figure 2 shows the contribution of malaria to the total outpatient visits in each age group. Malaria accounted for almost 40% of the outpatient visits in children aged two years or older, but this percentage was lower in the first two years of life, during which it increased gradually with age.

**Figure 3:**
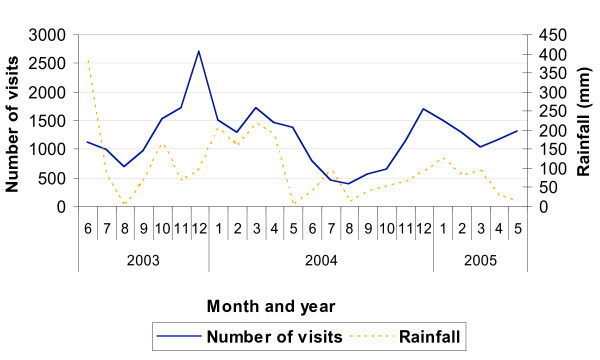
Seasonality of outpatient malaria visits and rainfall (mm).

Figure 3 presents the seasonality of malaria cases and the rainfall recorded. Sixty-three percent of the malaria cases occurred during the six months of rainy season (p < 0.0001), peaking in December each year, and the percentage of outpatient visits due to malaria was higher during the rainy than the dry season (32.0% vs. 29.4%, χ^2^(1 d.f.) = 69.5, p < 0.0001).

Table 1 summarizes the clinical characteristics of children with malaria presenting at the outpatient clinic. Among children who presented with malaria 56.7% had fever and 24.2% had hyperpyrexia. The median of reported days of fever was significantly higher in children who were transferred to the day-care unit (2 days, IQR 1–3 days) than among those who were sent home after being visited at the outpatient clinic (1 day, IQR 1–2 days, p < 0.0001). Children resident in the study area also had a shorter duration of reported fever (1 day, IQR 1–2 days) than children from outside the study area (2 days, IQR 1–3 days, p < 0.0001). On the other hand, only 37.2% of children with fever or a reported history of fever during the previous 24 hours had malaria parasites and were therefore diagnosed as malaria cases. Among those who presented with fever this percentage was 47.8% and among those who were afebrile on arrival but reported fever it was 21.2%.

**Table 1:** Anaemia, fever and parasitaemia among outpatient malaria cases

Mild anaemia (%, n/N)	41.3% (11783/28525)
Moderate anaemia (%, n/N)	11.9% (3407/28525)
Severe anaemia (%, n/N)	0.9% (249/28525)
Mean (SD) PCV	31.2 (6.0)
Axillary temperature ≥ 37.5°C (%, n/N)	56.7% (16413/93429)
Axillary temperature ≥ 39°C (%, n/N)	24.2% (7015/93429)
Median length (IQR)* of reported fever on arrival (days)	1 (1; 2)
Geometric mean parasitaemia (95% CI)^# ^(parasites/μL)	8582.8 (8346.3–8826.0)

*IQR: interquartile range

#95% CI: 95% confidence interval

**Figure 4:**
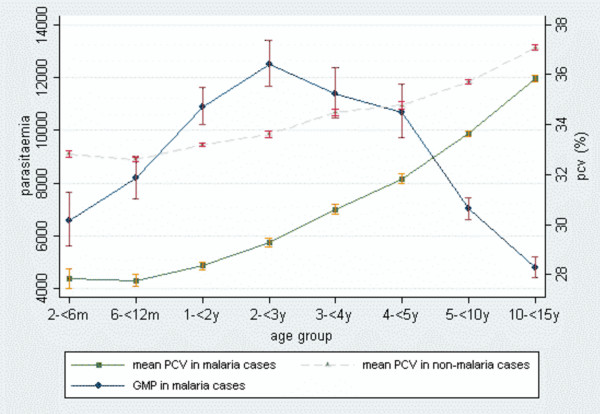
Geometric mean parasitaemia (GMP) and mean PCV (with 95% CI) among malaria outpatients and mean PCV among non-malaria outpatients by age group.

The geometric mean parasitaemia (GMP) increased with age during the first three years of life, peaking in children aged two to three years and then decreased gradually with age. Figure 4 shows the GMP and the mean PCV in malaria and non-malaria cases by age, including only children aged older than two months. The GMP in malaria cases was 8,582.2 parasites/μL, being significantly higher in children with fever (13,989.7) than in afebrile children (4,529.4, p < 0.0001) and in children with hyperpyrexia (24,003.3) than in those with a fever lower than 39°C (9,349.7, p < 0.0001). It was also higher among children transferred to the day-care unit (12,648.7) than among children sent home directly after being visited at the outpatient clinic (7,292.1, p < 0.0001). Among children transferred to the day-care unit, it was higher in children finally admitted to the wards (15,510.0) than in children sent home (9,860.3, p < 0.0001). GMPs were also higher during the rainy season (9,627.3) than during the dry season (7,074.5, p < 0.0001).

More than half of the malaria patients were anaemic, 11.9% had moderate anaemia, compared to 2.0% of the non-malaria cases (χ^2^(1 d.f.) = 3277.2, p < 0.0001), and 0.9% had severe anaemia, compared to 0.1% of the non-malaria cases (χ^2^(1 d.f.) = 242.1, p < 0.0001). The mean PCV was significantly lower in malaria cases (31.2%, SD 6.0) than in non-malaria cases (34.4%, SD 4.8, p < 0.0001). The mean PCV increased gradually with age both in malaria and non-malaria cases, although the increase was greater among malaria cases, in which the PCV ranged from 27.8% in infants aged 2 to 12 months to 34.4% in children aged 5 years or older (p < 0.0001 ANOVA and linear regression). The mean PCV difference between malaria and non-malaria cases was 4.9% (95% CI 4.7–5.1) in infants and 1.8% (95% CI 1.7–1.9) in children aged five years or above. The prevalence of moderate and severe anaemia presented no seasonality, being the same during the rainy and dry seasons both in malaria and total patients, although 63% and 60% of the total moderate and severe anaemia cases respectively occurred during the rainy season (p < 0.001).

Among malaria patients an inverse relationship was observed between PCV and density of parasitaemia. Among children younger than five years, doubling the parasitaemia was associated with a decrease in the mean of PCV of 0.14 units (%) on average, while among children aged five years or older, this decrease was of 0.07 units (p < 0.0001 linear regression, for both age groups).

Table 2 presents the outcome of outpatient malaria cases by age. Among children with malaria, 28.1% were transferred to the day-care unit, and of these 54.1% were finally admitted to the ward. These percentages and the percentage of children transferred to Maputo Central Hospital show a clear decreasing trend with age (p < 0.0001, test for trend). Overall, infants with malaria were admitted in a significantly higher proportion than children aged 1–4 years (33.4% vs. 19.0%; χ^2^(1 d.f.) = 344.7, p < 0.0001). Also, among malaria cases, children resident in the study area were transferred to the day-care unit and then admitted to the wards with a significantly lower frequency than children from outside the study area (23.6% vs. 33.9%, χ^2^(1 d.f.) = 371.7, p < 0.0001 and 45.4% vs. 62.0% of those transferred to the day-care unit, χ^2^(1 d.f.) = 225.1, p < 0.0001, respectively).

**Table 2:** Outcome of outpatient malaria cases by age

	After outpatient* (%)				After day-care unit* (%)		
Age group	N	Home	Day-care unit	Transfer**	Home	Admitted	Transfer^#^

<1 month	42	35.7	61.9	2.4	42.3	53.8	0
1–<6 months	1019	40.8	55.4	3.4	32.6	62.6	4.4
6–<12 months	2450	41.7	55.3	2.7	37.1	59.3	3.2
1–<2 years	5201	51.4	46.1	2.3	38.9	57.9	2.6
2–<3 years	4203	62.5	35.6	1.6	42.7	54.6	2.3
3–<4 years	3069	72.5	26.0	1.2	47.8	50.3	1.1
4–<5 years	2383	79.6	19.8	0.5	53.6	44.9	0.8
5–<10 years	6763	87.7	11.9	0.3	59.7	39.1	0.7
10–<15 years	3747	94.0	5.6	0.1	56.4	42.7	0.5

Total	28877	70.4	28.2	1.2	43.1	54.1	2.3

*Totals do not add up to 100%, as the percentage of children who absconded or died are not presented in the table

**Transfers mostly from Maragra Health Post to Manhiça District Hospital

^#^Transfers mostly from Manhiça District Hospital to Maputo Central Hospital

Minimum community-based incidence rates of outpatient malaria per 1000 CYAR for the whole study period were of 394 in infants, 630 in children aged 1 to <5 years and 237 in children aged five years and older. Table 3 presents the age-specific MCBIRs in the Manhiça study area by year. These incidences include only malaria cases in children resident in the study area. Among this group, there were 14,764 cases of malaria during the study period, experienced by 7,365 children. More than half of these (4,221/7,365, 57.3%) presented only once to the health centres with an episode of malaria, 19.9% (1,464) presented twice, 9.2% (674) presented three times and the remaining 13.6% presented four and up to a maximum of 14 times with malaria, showing a clustering of the clinical cases in a relatively small number of children. Considering that 26,923 study area children contributed to the time at risk, only 27.4% (7,365/26,923) of children in the study area have attended at least once the outpatient clinic with malaria during the two year study period. For children aged one to four years, who account for the majority of the malaria cases, this percentage is 33.2%.

**Table 3:** Age-specific minimum community-based incidence of clinical malaria, events per child years at risk

	June 2003–May 2004				June 2004–May 2005			
Age group	Episodes	CYAR	Rate per 1000 CYAR	95% CI	Episodes	CYAR	Rate per 1000 CYAR	95% CI

<1 month	8	155	51.7	25.9–103.4	7	153	45.8	21.9–96.2
1–<6 months	252	788	319.9	282.7–361.9	112	767	146.0	121.3–175.7
6–<12 months	700	852	821.5	762.8–884.6	334	871	383.6	344.6–427.1
1–<2 years	1490	1539	968.3	920.4–1018.8	842	1679	501.6	468.8–536.6
2–<3 years	1223	1410	867.6	820.3–917.6	899	1549	580.6	543.8–619.8
3–<4 years	1030	1435	717.6	675.1–762.8	664	1448	458.5	425.0–494.8
4–<5 years	868	1439	603.1	564.3–644.6	533	1484	359.2	330.0–391.1
5–<10 years	2279	6648	342.8	329.0–357.2	1701	6973	244.0	232.6–255.8
10–<15 years	1043	5265	198.1	186.4–210.5	779	5606	139.0	129.5–149.1

CYAR: child years at risk

95% CI: 95% confidence interval

The incidence of malaria in children aged 1 to 4 years was significantly higher than in infants (RR 1.5 for the first year and 1.9 for the second year of the study, p < 0.0001 for both), whereas the incidence in children aged 5 years or older was lower than in infants (RR 0.5 for the first year and 0.8 for the second year of the study, p < 0.0001 for both).

The incidence in the first year of the study period was significantly higher than that during the second year (RR 1.6, p < 0.0001), this difference being highest in infants (RR 2.1, p < 0.0001).

## Discussion

This paper describes the characteristics of children presenting with clinical malaria to the outpatient clinic of two health facilities in a rural area of southern Mozambique. Malaria represents a huge burden for the primary health care services, accounting for around 30% of the total visits. This implies a considerable health service utilization derived from one single disease, which is likely to translate into a significant health expenditure.

The age distribution of the cases is typical of an area of moderate transmission, with most of the cases occurring in children younger than 5 years, with a peak in children aged 1 to 2 years [15]. Despite malaria not representing a high percentage of the total outpatient visits among children younger than three years, this age group carries most of the burden of disease, accounting for around half of the total malaria cases.

The age distribution of outpatient malaria is more spread out than that of malaria requiring admission to the wards, as children younger than three years represent 45% of the cases among outpatient attendees, while children younger than two years account for 58% of the cases among admitted children [8]. Interestingly, children aged 5 to 14 years represent 36% of the malaria outpatient cases, whereas this percentage is only 9% among malaria cases that require admission to the hospital [8]. This supports the hypothesis that immunity against severe malaria develops faster than against milder forms [16], as the percentage of children in this age group that need admission is low. Nevertheless, it also shows that malaria in older children and teenagers has been previously underestimated and that malaria control programmes should also target them [17]. Control tools that would be of use for this age group include insecticide-treated nets, although distribution systems should be developed, and new control strategies such as school-based intermittent preventive treatment (IPT). Data from research conducted in Africa in the 1950s suggest that continuous chemoprophylaxis in schoolchildren does not impair the development of naturally acquired immunity and that it does not cause a rebound effect afterwards. Nevertheless, to avoid development of drug resistances and facilitate sustainability, intermittent preventive treatment, that could be maybe delivered at school level, would be a better option [18].

Infants, who suffer an important incidence of malaria, are transferred to the day-care unit and admitted to the wards with a significantly higher frequency than older children, reflecting either a greater severity of disease in infants, a high co-morbidity and/or a tendency of the medical staff to admit them more easily. Malaria control strategies should target children younger than three years, the age group most at risk, and particularly infants, who carry a disproportionate amount of severe disease.

Presentation of malaria cases to the outpatient clinic showed marked seasonality, with a peak in December each year. This seasonality is related to the actual rainfall, but the peak in malaria cases does not always follow the peak in the rainfall, as shown in Figure 3, suggesting there are other factors affecting the health facility-based malaria incidence, including health-seeking behaviour patterns.

According to the Integrated Management of Childhood Illness, children with a measured or reported fever in high malaria risk areas, and in the absence of facilities equipped with a microscope, should be classified as having malaria and receive presumptive antimalarial treatment [19]. In this study, only 37% of children who presented to the outpatient clinic with fever or a history of fever had malaria, reflecting the low specificity of fever (measured or reported). In a health post without a microscope or rapid diagnostic tests, 63% of children would be treated for malaria unnecessarily and probably not treated for the underlying condition, increasing the risk of severe disease, resistances to antimalarials and adverse effects. In the context of artemisinin-combination therapies (ACTs), often used presumptively as first line antimalarials, safety issues and cost have to be taken into account, and the risk of giving drugs unnecessarily should not be underestimated. Strengthening of health services through the availability of rapid diagnostic tests or microscopic facilities is crucial to decrease presumptive treatment and slow the rate of development of resistances. Concerns have been raised about the drug pressure that IPTi would exert if implemented widely, however presumptive treatment of malaria based on clinical symptoms (mainly presence of fever) is probably a considerable and greater source of drug resistance. The median duration of reported fever was one day, showing that in this area most families do not wait very long to go to hospital. Nevertheless children who were transferred to the day-care unit reported a longer, although still very short, duration of fever. This, together with the fact that children coming from outside the study area reported a longer duration of fever and were transferred to the day-care unit and admitted to the wards more frequently, reflects the fast evolution of malaria, with severity increasing with time without treatment.

Only 57% of children with malaria presented with a measured fever, which indicates that if measured fever was used as the only criterion to collect a blood slide many cases would not be detected and treated.

Parasite density was higher in admitted children, probably reflecting that high parasite levels on its own are very often a criterion for admission, especially when children are seen by a medical agent and not by a physician. Nevertheless, the level of parasitaemia – although it was associated with lower PCVs – was not found to be a risk factor for severity of malaria neither in admitted children in this area [8] nor elsewhere [20-22].

The decrease of parasitaemia density and the increase of the mean PCV with age reflect the development of naturally acquired immunity against malaria. The peak of the incidence of clinical malaria is in children aged one to three years, and coincides with the peak in parasitaemia density, that occurs in children aged two to three years. This argues against the widely accepted hypothesis that anti-disease immunity is different from anti-parasite immunity and supports the idea that actually these two immunities are the same and develop at the same time [23]. In an active case detection study conducted in the area the peak parasite density was at age one year, pointing in the same direction [6].

More than half of malaria cases present with anaemia, being moderate or severe in 13% of the cases. The aetiology of anaemia in Manhiça and the relative contribution of malaria to its development have not been determined, but malaria appears to be an important contributor, as children with malaria have a significantly lower PCV than children without malaria. This difference in the mean PCV between malaria and non-malaria cases is greater in younger than in older children, suggesting that malaria is a greater contributor to anaemia in infants and young children [24]. This is in agreement with chemoprophylaxis and bed net studies, where it was also observed that efficacy against anaemia was higher in younger infants [18,25]. On the other hand, the lack of significant seasonality of the prevalence of anaemia suggests that there are other causes of anaemia-nutritional or other infections – that are also present during the dry season.

The malaria MCBIR in children younger than five years from Manhiça, 575.7 per 1,000 CYAR, is within the interval of the estimated incidence rate for that age group in Africa in 2000 for rural areas with an EIR < 100 infective bites per person per year (414 – 1924 per 1000 CYAR) [26]. The malaria minimum community-based incidence rate in infants is relatively low during the first six months of life but increases in children aged six to 11 months, and peaks in children aged one to three years. A malaria active case detection (ACD) study conducted in children younger than 10 years in the study area during the period 1996–99 found a similar age pattern, with the peak incidence of clinical malaria being between six months and three years [7]. Infants in this area start experiencing malaria very early in life. During the first year of life the MCBIR increases with age, probably a function of a decreased exposure to mosquitoes in small infants, the protective effect of foetal haemoglobin in the first weeks of life, and the presence of maternally-acquired malaria-specific antibodies, even though the protection they confer is still a matter of debate [27]. However, the almost negligible incidence rates observed in newborns are probably underestimates of the real burden of the disease, as newborns being born at the hospital and becoming ill are often directly admitted from the maternity ward, and the collection of data as well of slides through the outpatient department may have been suboptimal during the study period.

The MCBIRs show a 40% decrease from the first year to the second year of the study. This reduction in the incidence shifted the age distribution to the right, being this decrease highest for infants. The rate ratio of the incidence in children aged one to four years old versus infants was highest in the second year. This change also illustrates the interannual variability in malaria incidence, which can be due to multiple reasons, discussed more thoroughly in the second part of this article [8].

More than 70% of the study area children have never presented to the health facilities with malaria, and among those who have, more than half had only one episode. These results are very similar to those found during the active case detection study, where 71% of children never had malaria and 50% of those who did, only had one episode, and to the results of the control group of the RTS, S malaria vaccine trial conducted in the area, where these percentages were even higher (80.5% and 70% respectively) [28]. This supports the idea that there is a clustering of the clinical episodes, whereby many children have no malaria, some have just a few episodes and very few have many episodes, appearing to be at a greater inherent risk than others.

## Conclusion

This article presents a retrospective analysis of outpatient data on malaria in children. Despite the limitations of hospital-based data, these provide an indication of the burden and age pattern of the disease, that may guide policy makers. Children younger than three years carry the burden of malaria, and control strategies should be targeted at them. Children between five and 15 years also suffer an important proportion of the disease, although less severe, and should be included in control programmes. Moreover, this would also decrease the enormous toll paid by health facilities to manage this disease. Preventive measures together with effective treatment of correctly diagnosed cases should on the long term guarantee that malaria ceases to be the number one cause of childhood disease in Africa.

## Authors' contributions

The hospital surveillance system and the DSS in Manhiça were designed by JJA, CM and PLA. They were set up in 1996 and have received numerous contributions to its design and implementation since then. During the study period QB, EM, PA, BS, AB, JS and TN were involved in the diagnosis and management of malaria patients and collection of data. AN coordinates the DSS in Manhiça. CG and QB led the analysis, interpretation and write up of this data set, and received input from all authors. All authors read and approved the final manuscript.
